# A randomized controlled trial of 3% polidocanol foam with saline prewashing compared with standard foam sclerotherapy for the treatment of varicose veins

**DOI:** 10.1016/j.jvsv.2026.102495

**Published:** 2026-04-01

**Authors:** Lidiane Aparecida Ferreira Rocha, José Ben-Hur Ferraz Parente, Viviane Santana da Silva, Isabela Zampirolli Leal, Marcela Pereira Zanoni, Alexia Paganotti, Giuliano Giova Volpiani, Eduardo Ramacciotti

**Affiliations:** a Santa Casa de São Paulo School of Medical Sciences, São Paulo, SP, Brazil; b Division of Vascular and Endovascular Surgery, Israelita Hospital Albert Einstein, São Paulo, SP, Brazil; c Science Valley Research Institute, São Paulo, SP, Brazil; d Hemostasis & Thrombosis Research Laboratories, Loyola University Medical Center, Maywood, IL

**Keywords:** Varicose veins, 3% polidocanol, Foam protocol, Sclerotherapy

## Abstract

**Background:**

Chronic venous disease is a highly prevalent global condition. Sclerotherapy with polidocanol foam is an established minimally invasive treatment. However, complications such as postprocedural hyperpigmentation remain common, and the maximum injectable volume per session may limit treatment efficacy. These factors highlight the need for optimized techniques to enhance vein occlusion and cosmetic outcomes. This study evaluated the efficacy and safety of 3% polidocanol foam with prewashing with saline compared with the conventional 1% polidocanol foam method.

**Methods:**

This single-center, prospective, randomized controlled trial enrolled 35 patients (70 limbs) with Clinical Ethological Anatomical Pathophysiological class C2 to C5 varicose veins. Participants were randomized 1:1 to receive either 3% polidocanol foam with 0.9% saline prewashing and extrinsic compression using a low-density polyethylene cylinder at 20 to 30 mm Hg, or 1% polidocanol foam. Primary outcomes were venous occlusion at day 90 on Doppler ultrasound examination and foam volume. Secondary outcomes were superficial venous thrombosis, hyperpigmentation, and quality of life, assessed using the revised Venous Clinical Score at baseline and day 90.

**Results:**

Thirty-one patients (62 limbs) completed the study (16 in the 3% polidocanol plus saline prewashing group; 15 in the 1% polidocanol group). Baseline characteristics were comparable across treatment arms. No major adverse events occurred. Occlusion rates were similar between the groups (*P* = .80). The 3% polidocanol foam technique achieved comparable occlusion using significantly less foam volume (0.27 ± 0.21 mL vs 0.54 ± 0.28 mL; *P* < .001). Rates of superficial thrombosis and hyperpigmentation were low and did not differ significantly. No statistically significant difference in the revised Venous Clinical Score was observed between groups; the small sample size may limit interpretation of these findings.

**Conclusions:**

The 3% SALFOAM method can be used with comparable efficacy and safety while using a lower sclerosant volume.

**Article Highlights**

• **Type of Research:** Prospective randomized study
• **Key Findings:** Treatment with 3% polidocanol foam plus saline prewashing, compared with standard 1% foam, in 31 patients with varicose veins, resulted in similar occlusion rates with lower sclerosant foam volume. Both techniques led to low rates of superficial thrombosis and hyperpigmentation.
• **Take Home Message:** The 3% polidocanol foam plus saline prewashing method can be used with comparable efficacy and safety, requiring less sclerosant volume.

Chronic venous disease affects nearly one-half the global population, with a higher prevalence in women (50.9%) than men (37.9%). It ranges from cosmetic telangiectasias to venous ulcers, representing a significant socioeconomic burden globally.^1^ The Clinical Ethological Anatomical Pathophysiological (CEAP) classification system, established by the American Venous Forum,^2^ standardized disease description, enabling better clinical communication and management planning.^3^

Among minimally invasive therapies, polidocanol foam sclerotherapy has become an essential treatment modality.^4^ The technique induces endothelial injury, resulting in thrombosis, fibrosis, and vessel occlusion; however, residual hyperpigmentation remains a common drawback.^5^^,^^6^ In randomized clinical trials evaluating foam sclerotherapy of the great saphenous vein, average volumes per session are approximately 4 to 5 mL, with the total foam volume rarely exceeding 10 mL per session.^7^^,^^8^

Historically, sclerotherapy has evolved from crude chemical irritants used since the seventeenth century to the modern use of polidocanol. The development of microfoam in 2001 by Cabrera and subsequent refinement by Tessari established contemporary practice.^9^^,^^10^

The effectiveness of sclerosing agents in axial veins is lower than that observed with endovenous ablative therapies. In follow-up periods of ≤5 years, thermal techniques maintain greater anatomical durability and better disease-specific quality-of-life outcomes than foam, which is why its use in axial veins is recommended selectively and dependent on the clinical and anatomical context.^11^^,^^12^

In Brazil, Evangelista^13^ and Ceratti^14^ introduced echo-guided foam sclerotherapy. Subsequent clinical trials confirmed its efficacy in ulcer healing and in improving quality of life.^15^, ^16^, ^17^ International guidelines reaffirm the technique's safety and role in varicose vein management.^3^^,^^4^

Randomized studies comparing 1% and 3% polidocanol in foam sclerotherapy have not demonstrated a significant difference in anatomical outcomes in medium- and long-term follow-up. In double-blind clinical trials with follow-up of ≤3 years, the final rates of abolition of great saphenous vein reflux were similar between the two concentrations when additional treatments were allowed,^7^^,^^18^ with a comparable safety profile.^19^

Despite these advances, procedural refinements that may reduce adverse effects and improve outcomes remain desirable. The proposed 3% polidocanol foam technique (named SALFOAM, an acronym for SALine plus FOAM 3% plus extrinsic compression maintained for 72 hours) introduces a prewashing with saline solution, the use of a higher concentration of foam (3%), and extrinsic compression using a low-density expanded polyethylene cylinder, aiming for efficient endothelial contact with reduced sclerosant volume. This study was designed to compare the SALFOAM 3% technique with the conventional 1% polidocanol foam (both using the Tessari 4:1 mixing method), looking at venous occlusion rate measured by Doppler ultrasound examination at day 90, the volume of sclerosant used, and complications (superficial thrombosis, hyperpigmentation) in the treatment of lower limb varicose veins.

## Methods

### Study design

This was a single-center, prospective, randomized, controlled, parallel-arm clinical trial with per-limb analysis. Conducted at the Vascular Surgery Outpatient Unit, Hospital Sao Luiz Gonzaga—Santa Casa de Misericordia de Sao Paulo. The study adhered to the CONSORT 2010 and SPIRIT 2015 recommendations.^20^, ^21^, ^22^

### Ethics considerations

This study was approved by the Research Ethics Committee of Santa Casa de Misericordia de São Paulo (CAAE 6565.1422.60,000.9147; Opinion No. 5.920.068) and registered at ClinicalTrials.gov (NCT06667570). All participants provided written informed consent. The study complied with the Declaration of Helsinki.

### Participants

The study enrolled patients of both sexes with CEAP class C2 to C5 varicose veins. Inclusion criteria were age >18 years, CEAP class C2 to C5 disease, bilateral tributary varicose veins ≥3 mm diameter, ≥10 cm in length, and signed informed consent. Exclusion criteria included pregnancy, allergy to polidocanol, infection, immunosuppression, coagulopathy, prior venous thromboembolism, patent foramen ovale, or refusal to participate.

### Randomization and blinding

Randomization (1:1) was performed via REDCap,^23^ with allocation concealment. Statisticians and outcome assessors were blinded to group assignments.

### Interventions

#### SALFOAM 3% method

Venous puncture using a 25G scalp needle (Becton, Dickinson and Company) was accomplished, Washing with 0.9% saline and injection of 3% polidocanol foam (Victalab Indústria e Comércio de Produtos Quimicos) was prepared by Tessari 4:1 technique using 3-mL syringes (Terumo Corporation), followed by manual drainage and immediate extrinsic compression with a 1-cm foam plaster (LIDstop Industria de Produtos Hospitalares). Then, 20 to 30 mm Hg compression stockings were applied (Medi GmbH & Co) for 72 hours.

#### Standard 1% polidocanol method

Standard 1% polidocanol foam was injected (Victalab Industria e Comercio de Produtos Quimicos) at a 4:1 ratio, also by the Tessari method. Dental cotton rolls (Cremer S.A., Blumenau) were used for compression, along with 20 to 30 mm Hg compression stockings for 24 hours plus 5 daytime days.

Only tributary varicose veins of the lower limbs were included. The randomization unit was the individual, including both lower limbs, provided that each leg presented at least one eligible vein. In each limb, a single target vein was defined, previously chosen by clinical evaluation and direct inspection, regardless of the presence of other varicose veins in the same limb. A single target vein (standardized segment) was selected for comparative analysis in the protocol (isolated tributary vein, diameter ≥3 mm and with ≥10 cm in length, in bilateral lower limbs).

The injection was performed directly into the target vein, without ultrasound guidance, because these superficial tributary veins were easily accessible. The target vein was documented by ultrasound examination before the procedure, and follow-up was performed with ultrasound imaging, with only this vein being considered for analysis in the study outcomes.

Prewashing was performed by intraluminal injection of saline solution, without a predefined volume, titrated until visual clearance of the tributary vein or until progression was mechanically limited by venous spasm; the foam was injected immediately afterward, and titrated to the technical end point. Because the protocol was restricted to superficial tributary veins, ultrasound monitoring was not used for the washout. Two different vascular surgeons each performed only one of the two techniques, ensuring procedural consistency and minimizing operator bias.

#### Photographic documentation

Photography was performed under a rigorously standardized protocol to ensure reproducibility. The images were obtained in a room without natural lighting, using exclusively indirect lighting from two fixed reflectors, maintained in the same position and with constant intensity throughout the study. An infinite white background suitable for clinical photography was used. The distance between the participant and the photographer was standardized to 1 m, with floor markings to ensure identical positioning in all assessments. The same image capture device (iPhone 16 Pro Max) was used in all sessions. Six standardized positions were performed: anterior, posterior, right oblique at 45°, and left oblique at 45°, in addition to a specific recording of the target vein in each lower limb. Photographs were obtained on day 0 and repeated on day 90 under identical conditions, including lighting, positioning, and framing. The target vein was identified in a standardized manner in the comparative images.

#### Image analysis

The process followed these steps: the same comparative images of the treated areas, collected on days 0 (initial) and 90 (final), were used for each participant. The photographs were converted to grayscale, and a standardized region of interest was delineated using the rectangular tool in ImageJ. The following parameters were extracted: area (in pixels), average gray value, and minimum gray value. The data were recorded individually in REDCap for subsequent statistical analysis.

To standardize visual analysis and ensure blinding, a comparative montage was created in Canva. This montage included photos from days 0 and 90, taken in a standardized fashion, positioned side by side, with the delimitation of the treated area in each limb. The montages were inserted into an electronic form (Google Forms). The evaluation form did not include any information about participants' clinical indications or the type of treatment performed, thus ensuring evaluator impartiality. The evaluators received standardized instructions to classify the degree of hyperpigmentation using a visual scale with four levels: 1 = absent—skin without any evidence of residual pigmentation; 2 = light pigmentation—very discreet, only perceptible with close attention; 3 = moderate pigmentation—visible but with a light tone; and 4 = intense pigmentation—dark and evident, with a brownish tone.

### Follow-up and data collection

Assessments occurred at D0, D+7, D+30, and D+90. Data included demographic parameters, CEAP and Fitzpatrick classification,^19^ ultrasonographic evaluations (Shenzhen Mindray Bio-Medical Electronics Co.), and photographic documentation under standardized lighting in a photographic studio.

### Outcomes

#### Primary efficacy outcome

The primary efficacy outcomes were venous occlusion on ultrasound and the volume of sclerosant used. The volume of sclerosant used for each treatment was recorded in milliliters.

#### Primary safety outcome

Safety outcomes included superficial axial vein thrombosis, hyperpigmentation, and allergic reactions. Objective pigmentation analysis was conducted using ImageJ software.^24^

#### Secondary outcomes

Secondary outcomes included the revised Venous Clinical Severity Score quality-of-life questionnaire administered at days 0 and 90 of follow-up.^25^

##### Statistical analysis

No sample size calculation was carried out. Because the primary outcome incidence was unknown for the 3% polidocanol foam, a convenience sample of 35 patients was selected. Analysis was performed using RStudio (RStudio, PBC) and GraphPad Prism 5 (GraphPad Software). Continuous variables were expressed as mean ± standard deviation, and categorical variables as frequency (%). Between-group comparisons employed Fisher's exact, χ^2^, Welch, or Mann-Whitney *U* tests as appropriate. Interobserver agreement was measured using Fleiss' Kappa. Regression analyses and Spearman correlations were used for associations. Significance was set at a *P* value of <.05.

## Results

From October 2024 to January 2025, 110 eligible patients were screened; 35 were included, and 31 completed the study (CONSORT diagram) (Fig). Thirty-one participants (62 limbs) were analyzed: 16 in the SALFOAM 3% group (32 limbs) and 15 in the standard 1% polidocanol foam group (30 limbs). Groups were homogeneous with respect to age, body mass index, and sex distribution (*P* > .05). The majority were female (81%) with a mean age of 60.9 ± 12.0 years. CEAP class C2 disease predominated in both groups (56% vs 80%; *P* = .30). Fitzpatrick phototypes III and IV were most frequent, with no intergroup differences (*P* = .91) (Table I).

**Fig:**
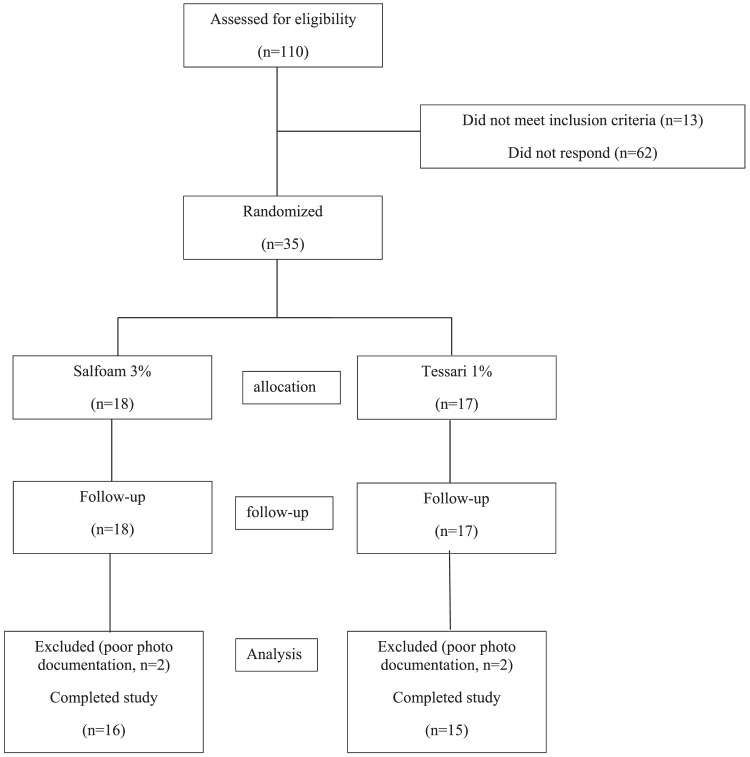
CONSORT diagram.

**Table I:** Baseline characteristics

Characteristic	SALFOAM 3% (n = 16)	Polidocanol 1% (n = 15)	*P* value
Age, years	62.3 ± 11.3	59.5 ± 12.9	.529
Weight, kg	77.5 ± 12.9	73.7 ± 12.6	.417
Height, m	1.61 ± 0.05	1.63 ± 0.10	.580
Body mass index, kg/m^2^	29.8 ± 4.2	28.2 ± 5.6	.351
Female sex	13 (81.3)	12 (80.0)	1.000^a^
CEAP clinical class			
C2	9 (56.3)	12 (80.0)	
C3	3 (18.8)	2 (13.3)	
C4	4 (25.0)	1 (6.7)	
C5	0 (0.0)	0 (0.0)	
Fitzpatrick skin type			
II	1 (6.3)	0 (0.0)	
III	8 (50.0)	6 (40.0)	
IV	5 (31.3)	5 (33.3)	
V	2 (12.5)	3 (20.0)	
VI	0 (0.0)	1 (6.7)	

*CEAP*, Clinical Ethological Anatomical Pathophysiological.

Values are mean ± standard deviation or number (%).

a Fisher's exact test.

Venous occlusion rates were similar between SALFOAM 3% (50%) and the standard 1% polidocanol foam group (53.8%) (*P* = .80). Both groups demonstrated comparable proportions of sclerotic vs thrombotic occlusion patterns (72.2% vs 64.3%; *P* = .71). These results are detailed in Table II.

**Table II:** Primary, secondary and safety outcomes

	SALFOAM 3%	Polidocanol 1%	*P* value
Primary efficacy outcome—venous occlusion			
Occlusion			
Yes	18 (50)	14 (53.8)	.802
Type of occlusion (among occluded vessels), %			
Sclerosis	72.2	64.3	.712
Thrombosis	27.8	35.7	
Primary efficacy outcome—sclerosant volume			
Target vessel diameter, mm			
3.0-5.0	29 (80.6)	23 (88.5)	.575^a^
5.1-7.0	3 (8.0)	2 (7.7)	n/a
≥7.1	4 (11.1)	1 (3.8)	n/a
Injected foam volume at D0, mL			
Mean ± SD	0.27 ± 0.21	0.54 ± 0.28	<.001^b^

rVCSS category	SALFOAM 3% D0	SALFOAM 3% D90	Polidocanol 1% D0	Polidocanol 1% D90	*P* value
Secondary efficacy outcome					
rVCSS severity^c^					
Mild (1-8)	55.6	76.5	93.8	94.1	.019
Moderate (9-15)	44.4	23.5	6.3	5.9	.335
Severe (>15)	0	0	0	0	—

	SALFOAM 3%	Polidocanol 1%	*P* value
Safety outcomes			
Hyperpigmentation grade			
Absent	13 (37.1)	10 (43.5)	
Mild	8 (22.9)	5 (21.7)	
Moderate	10 (28.6)	4 (17.4)	
Severe	4 (11.4)	4 (17.4)	.747^d^
	*R*	*P* value	
Spearman correlation with hyperpigmentation			
Target vein diameter (D0)	−0.00	.708	
Target vein diameter (D90)	−0.06	.655	
Fitzpatrick skin type	−0.22	.093	

*rVCSS*, Revised Venous Clinical Score; *SD*, standard deviation.

Values are number (%) unless otherwise indicated.

a *P* values refer to the global comparison across all diameter categories using Fisher's exact test.

b *P* < .001 refers to statistical significance of foam volume in the Tessari group only (regression model vs reference).

c The rVCSS was calculated as an absolute numerical score (0-30) at D0 and D90. Patients were subsequently classified into severity categories. *P* values refer to global comparisons of ordinal distribution between groups.

d *P* value refers to the overall comparison of hyperpigmentation distribution between groups.

The SALFOAM 3% method required significantly less foam than the standard 1% polidocanol foam method (0.27 ± 0.21 mL vs 0.54 ± 0.28 mL; *P* < .001). Stratification by venous caliber confirmed that smaller sclerosant volumes achieved comparable occlusion in SALFOAM-treated veins (Table II).

The frequency of hyperpigmentation, graded by blinded evaluators, was statistically similar between groups (*P* = .75). The overall Fleiss' kappa for agreement was 0.409, indicating moderate concordance. Objective ImageJ measures supported these findings, with no significant differences in mean gray value or pigmentation area. A complementary hyperpigmentation analysis assessed whether larger vessels and higher phototypes in the Fitzpatrick classification would result in greater hyperpigmentation than smaller vessels. None of these variables showed a statistically significant correlation with hyperpigmentation by Spearman correlation analysis. These results are detailed in Table II.

No serious adverse events occurred in either group. No neurological, respiratory, or anaphylactic reactions were recorded. The SALFOAM 3% technique resulted in no superficial thrombosis events; two minor cases occurred in the standard 1% polidocanol foam arm (*P* = .109). These results are detailed in Table II.

Quality of life, assessed by the revised Venous Clinical Severity Score, did not improve statistically from baseline to day 90, nor were there intergroup differences (Table II).

## Discussion

This trial demonstrates that the SALFOAM 3% method is a safe and effective alternative to conventional 1% foam sclerotherapy, achieving equivalent venous occlusion with a significantly smaller sclerosant volume.

The results align with prior evidence supporting foam sclerotherapy as an effective treatment for chronic venous disease.^26^ The reduction in polidocanol volume in the SALFOAM group likely reflects the presaline wash's mechanical preparation of the endothelium and improved foam distribution within the target vessel. Extrinsic compression may further enhance contact time, thereby promoting endothelial denudation and fibrosis.^27^

Safety outcomes were favorable: no serious adverse effects occurred, and only mild, self-limited complications were observed, consistent with prior studies. The absence of significant differences in hyperpigmentation incidence indicates that the higher sclerosant concentration did not exacerbate this effect.^5^

The SALFOAM technique, with its distinctive combination of steps (saline lavage, 3% polidocanol foam, and immediate compression), presents a practical, low-cost improvement to standard care. Importantly, its procedural simplicity supports scalability in outpatient practice. The injection end point was pragmatically defined for the target segment: the injection was stopped when the expected local clinical response was achieved (visible filling/whitening of the treated segment) and/or when it was not possible to progress with the injection owing to mechanical limitation/vessel spasm (more evident after prewashing with saline in the 3% group). This design explains smaller volumes without invalidating the rationale of the study, because the objective was to compare standardized therapeutic protocols in a controlled segment and not to reproduce a complete varicose vein treatment session.

A primary limitation is that the SALFOAM 3% approach used a predefined therapeutic protocol combining prior saline prewashing, 3% polidocanol foam, and a specific extrinsic compression strategy, whereas the control group followed the conventional protocol with 1% polidocanol foam and standard local compression. Therefore, the findings should be interpreted as the effect of an integrated procedural strategy rather than the isolated impact of a single component. It is not possible to determine the independent contribution of the sclerosant concentration, saline prewashing, or compression technique to the observed outcomes. Future studies with factorial or component-based designs will be needed to isolate the relative effect of each element. Additionally, the fact that a specific physician performed each technique may represent a potential source of performance bias. Although both professionals are experienced in foam sclerotherapy, individual differences in technique execution or decisions during the procedure may have influenced the observed results. Limitations also include the single-center design, small sample size, lack of blinding in assessors, and 90-day follow-up, which limit generalizability and long-term outcome evaluation. Photographic evaluation, although standardized, retains a subjective component despite blinded assessment. Larger multicentric trials with extended follow-up are required to confirm the durability of occlusion and hyperpigmentation outcomes.

Regardless of its limitations, this study is, to the best of our knowledge, the first to demonstrate that a practical, simple, and inexpensive protocol may be as safe and effective as the classic 1% polidocanol foam method, while using a smaller sclerosant volume.

This protocol may represent a reproducible improvement in foam sclerotherapy. The results presented allow for the structuring of a future study adequately sized to confirm the reduction in volume without loss of efficacy in terms of occlusion and to evaluate, with statistical robustness, the clinical impact on hyperpigmentation and safety. At the same time, this pilot raises additional relevant questions, particularly regarding the relative contribution of the different components of the protocol (sclerosing agent concentration, prior saline flushing, and compression strategy) and the possible relationship between lower volume used and pigmentary outcomes. Future multicenter and long-term studies are recommended to validate these findings and explore broader clinical applications.

## Conclusions

Prewashing with saline solution injection of 3% polidocanol foam, and extrinsic compression using a low-density polyethylene cylinder combined with 20 to 30 mm Hg compression (SALFOAM 3%) technique demonstrated safety and efficacy equivalent to the conventional 1% polidocanol foam method for the treatment of lower limb varicose veins, while requiring significantly less sclerosant volume.

## Declaration of generative AI and AI-assisted technologies in the manuscript preparation process

During the preparation of this work, the author(s) used Grammarly Version 14.1270 to check spelling, punctuation, and basic grammar errors. After using this tool/service, the authors reviewed and edited the content as needed and take full responsibility for the content of the published article.

## Author Contributions

Conception and design: LR, JP, VS, GV, ER

Analysis and interpretation: LR, JP, VS, IL, MZ, AP, GV, ER

Data collection: LR, JP, VS, IL, MZ, AP, ER

Writing the article: LR, JP, GV, ER

Critical revision of the article: LR, JP, VS, IL, MZ, AP, GV, ER

Final approval of the article: LR, JP, VS, IL, MZ, AP, GV, ER

Statistical analysis: LR, ER

Obtained funding: LR, JP, ER

Overall responsibility:LR

## Funding

No external funding was received. The study was entirely funded by the first author. This was a self-funded study, with modest financial support for data collection from the Science Valley Research Institute in São Paulo, SP, Brazil.

## Disclosures

E.R. reports grants and consulting fees from Bayer and Pfizer; research grants from Bayer, Althaia, Pfizer, Novartis, Bios, and the Brazilian Ministry of Science and Technology; and personal fees from Aché Pharma, EMS, Novartis, and Daiichi-Sankyo, outside the submitted work.
